# Dr. Edward Brown-Sequard’s Experimental and Clinical Researches—Epilepsy

**Published:** 1857-10

**Authors:** 


### SELECTIONS.

### *Dr. Edward Brown-Sequard's Experimental and Clinical Researches, applied to Physiology and Pathology.*

EPILEPSY—(Continued).

9TH. If there are contractions in the bloodvessels of the brain proper, as there are in the muscles of animal life, in the beginning of an epileptic seizure, it is very easy to explain the variety of sensorial and other cerebral symptoms of epilepsy. In the same way as there are *certain* muscles that contract in the neck, in the larynx, or elsewhere, we may admit that there are *certain* bloodvessels that contract either in some parts of the brain proper, or in the nervous portions of the organs of sense, and in consequence, there is a trouble or loss of either one or several senses, or of the intellectual faculties, consciousness remaining more or less entire; or there is a successive loss of sight, of hearing, of the intellectual faculties, and, at last of consciousness.

10th. It is well known that sometimes the compression of the carotid arteries stops a fit of epilepsy. Cases of this kind have been mentioned by Liston, Earle, Albers, etc. The same operation in certain animals, and particularly in rabbits in good health, is sometimes sufficient to cause convulsions, so that we are led to the question, How can the same circumstance in one case cause convulsions, and in another diminish or destroy them? My theory may give an explanation of this apparent opposition. Changes in the quantity of fluid in the cranio-spinal cavity cannot take place suddenly, and if there is a considerable diminution in the quantity of blood which enters this cavity, as is the case when the carotid arteries are compressed, there is necessarily a corresponding diminution in the quantity that goes out. The blood which reaches the encephalon by the vertebral arteries having to fill a much larger space, circulates more slowly and becomes much more charged with carbonic acid, and, besides, furnishes much less oxygen to the encephalon, so that if the compression of the carotid arteries be made in healthy animals, it causes convulsions, just as I have found that blood much charged with this acid injected into the carotid arteries, causes convulsions; whereas, if the compression of these arteries be made in man, during an epileptic seizure, there is at first usually a momentary increase in the intensity of the fit, and sometimes after one or two minutes, rarely sooner, a diminution in the violence of the convulsions, and in some cases, a complete cessation of these contractions. Those who have observed what takes place in animals when they are asphyxiated, have remarked that after violent convulsive struggles, while the blood is becoming more and more charged with carbonic acid, there is a diminution of the convulsions, and at last nothing but rare respiratory efforts. Carbonic acid, after having excited the vital properties of the nervous system, seems to destroy them gradually, allowing for a time, however, the production of respiratory movements. The compression of the carotid arteries in epileptics, during a fit, induces a state of asphyxia greater than that already existing, and in so doing, diminishes the vital properties so much that there are no more convulsions. Respiration taking place* then, and the blood-vessels of the brain proper relaxing, the whole encephalon receives more oxygenated blood, and the patient recovers in the

* Of all the reflex phenomena, the regular inspiratory and expiratory movements are those which last the longest; it is so during agony resulting from any disease, it is so after chloroform or ether have been inhaled in large doses, it is so in asphyxia by hanging, drowning, etc., and it is so also in epilepsy.

same way, and by the same means that he does when the compression of the carotid is not employed in a fit.

The theory of epilepsy that we have arrived at from the examination of the phenomena of this disease, is not in opposition with any that we know; and, still more, we might easily show that is in harmony with the most important facts concerning the causes, the variations of the symptoms, the consequences and the treatment of this convulsive affection. We will merely point out, in addition to what we have related above, that the production of epilepsy by lead (which is an excitant of contraction in bloodvessels), by loss of blood, etc., and the important relations of epilepsy with intermittent fever, are facts in perfect harmony with our theory.

We must now say a few words, 1st, on the production of the change in the cerebro-spinal axis, which chiefly constitutes epilepsy (*i. e.*, the augmentation of the reflex excitability); 2d, on the production of the change of certain parts of the skin, mucous membrane, etc., which renders these parts capable of exciting epileptic seizures; 3d, on the mode of production of a fit of epilepsy from excitations springing either from a peripheric part or a central part of the nervous system; 4th, on the consequences of an epileptic seizure, and on the interparoxysmal state.

1st. The production of a change in the reflex excitability of the cerebro-spinal axis we think may take place in two different ways, one of which is a *direct* abnormal nutrition, as in syphilitic, scrofulous or rheumatic epilepsy, while the other is an *indirect* abnormal nutrition, due to some excitation from a peripheric or a central part of the nervous system. The *modus operandi* of such excitations we do not know positively, but very likely, in a number of cases, at least, it is through the bloodvessels of the cerebro-spinal axis that these excitations operate to change the natrition of this nervous axis. We have ascertained that many substances which act upon the spinal cord, either in increasing its reflex faculty (such are strychnia, morphia, etc.), or in diminishing it (such are belladonna, ergot of rye, etc.), produce their effect chiefly by their influence on the bloodvessels of this nervous centre. When they excite the bloodvessels to contract, they diminish nutrition, and cause paralysis; when they diminish the contractility of the bloodvessels, and therefore allow them to dilate, there is more blood in the spinal cord, and its nutrition is increased. Then the reflex faculty becomes greater, and irritations may cause convulsions. In animals and men, not having taken any of these substances, the reflex excitability of the cerebro-spinal axis may be increased in the following ways. An excitation on some part of the nervous system causes a contraction of the small bloodvessels of a part of the cerebro-spinal axis, and as the same quantity of blood still arrives by the various arteries in the cerebro-spinal cavity, it results that if the small ramifica tions of some arterial branches are contracted, the others receive more blood, so that nutrition, and, in consequence, the reflex excitability, augment in the parts to which they are distributed. But this is not likely to be the most frequent mode of increase of nutrition. We have found that when a vascular nerve is excited for a long while, the contraction of the bloodvessels after a certain time ceases, and a dilatation takes place, which lasts longer than the contraction, although the nerve is still excited: this is a paralysis by excess of action. In the nervous centres, very likely the paralysis of the bloodvessels supervenes also after considerable contractions, and in consequence of this paralysis, nutrition is increased in the parts of these centres where it exists, as we have found that nutrition is increased in the nerves and muscles of the face, when their bloodvessels are paralyzed. With the increase of nutrition in the nervous centres comes the augmentation of the reflex excitability, which seems to be the principal element of epilepsy.

Besides these causes, there is another of greater importance, which may exist when they do not: the nerve-fibres animating the bloodvessels of the parts of the cerebro-spinal axis where epilepsy has its seat, may be paralyzed as the nerve-fibres of the muscles of animal life are by a disease of some part of the nervous centres, and the consequence of this paralysis is necessarily an increase of nutrition and of reflex excitability. This is a fact which we have positively ascertained; the section of a lateral half of either the medulla oblongata or the spinal cord is the cause of paralysis of the bloodvessels of the cord on the same side, the consequence of which paralysis is that nutrition and the reflex excitability of the cord become much increased. When the spinal cord is cut across entirely, in mammals as well as in cold-blooded animals, the part separated from the encephalon has its bloodvessels paralyzed, and therefore dilated. Nutrition and the reflex excitability are soon much increased in this part, and it is sufficient to touch the skin or the mucous membrane of the genital organs, or of the anus, to determine violent spasms.* This cause of production of epilepsy, or at

* I had said, in a paper read last year at the *Academie des Sciences* of Paris (*Arch. Gen. de Med.*, Fev., 1856), that in making the autopsy of my epileptic animals, a congestion of the base of the encephalon and of the Gasserian ganleast of an increased reflex excitability, must exist in a very great degree in cases of tumors of the pons Varolii, or of the medulla oblongata, and if they do not cause this convulsive affection more often it is very probably because the moral and the emotional excitation of fits cannot act in many of these cases.

When an excitation coming from some peripheric nerve produces in the cerebro-spinal axis the change of nutrition which causes epilepsy, it is very likety that this excitation sometimes, if not always, acts otherwise than by producing a contraction of some bloodvessels. Whether this action is like those due to electricity or not, we cannot tell, but we think that an opinion which we had held for many years with Donders, and some other physiologists,* must be modified. This opinion is that all the nervous influences on nutrition, secretion, &c., either direct or by reflex action, act only in causing contractions or paralytic dilatations of bloodvessels. This view, which has been criticised with much ability by Prof. James Paget, in his admirable lectures on nutrition and on inflammation, seems to have been proved to be too absolute by the important researches of Prof. Ludwig and his pupils (see *Physiol. des Menschen*, von Donders, vol. i., p. 187–9, 1856), which appear to establish positively that there is another mode of influence of the nervous system, at least on certain glands ; an influence resembling that possessed by electricity in causing chemical combinations or decompositions.†

2d. The changes produced in peripheric parts, rendering them able to excite fits of epilepsy, consist more in alterations in the nature of the excitations that may spring from peripheric nerves than from an increase in the felt excitations coming from these nerves. We have shown already that in our animals the skin is not more sensitive in the parts of the face which are

---

glion is found; but I have ascertained since that in a great measure this congestion is a result of the fits and of the irritation of the skin of the face by pinching or otherwise, and not a circumstance preceding the first fit, and connected with the production of the increase of the excito-motory power of the skin.

* The same thing sometimes occurs in man. In a case of fracture of the spine, recorded by Dr. Knapp (N. Y. Journal of Medicine, Sept., 1851, p. 198), there was a paralysis of the abdominal limbs. A month after the accident, there were slight spasms in those limbs; in four months, the spasms became violent; on exposure to the cold air, or to a sudden touch, the muscles were thrown into the most violent agitation.

† Prof. Claude Bernard, in announcing recently his important discovery of the substance which in the liver gives origin to sugar, expresses himself very strongly in favor of this opinion. (*Gaz. Med. de Paris*, 1857, p. 202.)

capable of exciting fits than in the other parts of the face which have not that power (see § IV). In man, as we have also shown elsewhere (see § XI.) it is to the nature of the excitation, and not to the degree of pain, springing from some peripheric nerve, that we must attribute the production of the fits. The fact that excitations, starting from the periphery and causing fits, may not be felt, and the fact that when there are sensations accompanying these unfelt excitations, they may vary as to their kind, and sometimes be very feeble, certainly are important arguments to show that the real cause of the fit is something which is not felt. If the term *aura epileptica* had not been employed already to express the sensations which accompany the excitation of the fits, it would be well to employ it to name the unfelt excitation which is the real exciting cause.

In Inquiring into the nature of the unfelt aura, we find that very probably it is nothing but a violent excitation originating in the excito-motory nerve-fibres. Dr. Marshall Hall and Mr. Grainger have long ago imagined that there are nerve-fibres which are employed in reflex actions, and not in sensations and in voluntary movements; but they did not adduce direct facts to prove the correctness of their views. I have found many facts which seem to give the proofs hitherto needed that there are nerve fibres which are employed in exciting reflex actions, and which are neither sensitive nor capable of transmitting sensitive impressions to the encephalon. I have found also, that the excito-motory power, like the sensibility of nerves, varies in different parts of their length (see my *Experimental Researches applied to Physiology and Pathology*, New York, 1853, p. 98), and also in the same part, according to various circumstances.

Besides, I have ascertained that in certain parts where the excito-motory power seems not to exist, it may be generated, and become considerable. Now, as the fibres which have this power seem not to be sensitive, we understand why an excitation may originate from them, reach the nervous centres, produce the loss of consciousness and convulsions by a reflex action, without giving pain, or even any sensation. We may understand also that this reflex excitation may produce cramps by a reflex action in the muscles which are in the neighborhood of the starting point of the excitation, which cramps give rise to a pain wrongly considered as a primitive aura, although it is only a secondary and almost inefficient one.

With the view that in the very beginning of epileptic fits, caused by excitations coming from peripheric nerves, it is not the sensitive nerve-fibres, but only the excito-motory fibres which are in action, we can easily explain many facts. For instance, in my animals, the power of giving rise to fits, belonging to the cutaneous ramifications of nerves and not to their branches or trunks; in man, the absence of sensations, although there is an excitation from some peripheric nerves, as in the case of M. Pontier (see § IX., Case VII., and many others mentioned in § XI).

What the causes of the increase of the excito-motory power are, we cannot tell positively. We know, however, that some causes increase all the vital properties of nerve everywhere, and among these causes we will point out a paralysis of the bloodvessels, or the development of inflammation. But there are other causes of which we are ignorant; in my animals, for instance, there is but a slight increase in the vascularizatiou of the part of the skin which has the power of giving rise to fits, and this might be due to the pinching employed to irritate the skin.*

The changes taking place in the peripheric nerves, either in the skin, in the mucous membranes or in their trunks, when they become able to excite epileptic fits, may be produced by the influence of distant parts. For instance, in my animals, alterations of the spinal cord as low down as the *cauda equina* have sometimes been productive of the peculiar change in the face and neck which renders these parts able to excite fits. In man, tumors of the brain seem to have produced a similar change in one arm.

In my animals I cannot decide whether it is through some direct nervous influence upon the nutrition of the skin of the face and neck, or if it is through an indirect influence, and by means of the bloodvessels, that the spinal cord acts on this part. I have found that changes in nutrition occur in other parts of the head—such as the cornea—in rnimals upon which the section of a lateral half of the spinal cord has been made, but is this a direct or an indirect influence? I cannot decide. It is very well known that the sympathetic nerve in the abdomen may influence the nutrition of the eye through the spinal cord, but does the influence result from a change in the calibre of the

---

* We still maintain, however, as we have done for many years, that the influence of the nervous system on nutrition and secretion, either direct or reflex, is in a great measure due to the inflnence of nerves on the muscular layer of the bloodvessels. Even galvanism, in improving nutrition, we have proved to act partly in this way; it contracts the bloodvessels, and in so doing diminishes circulation and warmth. But after a certain time of violent contraction, the bloodvessels become paralyzed and dilated, so that more blood passes through them, and the temperature and nutrition are increased.

blood vessels of the eye, or is it a direct influence, like that of certain nerves on the salivary glands, according to the great discovery of Ludwig ?

As regards tumors of the brain, the important case of Odier (see § VIII., Case I.) seems to show that they may produce in the arm that peculiar change in peripheric nerves which renders them able to excite fits of epilepsy. But it is by far much more probable that it was not by an action of the brain, but through the irritation of the sensitive or excito-motory nerves of the scalp, or in consequence of the compression of the base of the encephalon, that the change of nutrition took place in the arm.

3d. In the two preceding sections I have examined how are produced the two organic causes of epilepsy ; *i. e.*, the increase of the reflex excitability of certain parts of the cerebro-spinal axis, and the increase in the excito-motory power of the peripheric nerves. I have now to say a few words on the mode of production of the most interesting phenomena of a complete fit of epilepsy.

The first phenomenon of such a fit is not always the same, and this explains why the best observers do not agree in this respect. Dr. Marshall Hall for a long while considered as the first symptom a distortion of the eye-balls and of the features, and he admitted as the second phenomenon a forcible closure of the larynx, and an expiratory effort (*Diseases and Disarrangements of the Nervous System*, 1841, p. 323). In many subsequent publications (see *Lancet*, June 12, 1847, p. 611, and *Aperçu du Systeme Spinal*, 1855, p. 201) he seems to consider as the first phenomona the contraction of the muscles of the neck and of the larynx. Dr. C. J. B. Williams (*General Pathology*, 2d Am. Ed., p. 166) says that the first phenomenon is a palpitation of the heart. Herpin (*Loco cit.*, p. 421-5) after having tried to show that when there is an aura the first phenomenon consists in a local cramp, says that the second phenomenon (the first when there is no aura) is the epilectic cry. According to Bean (*Arch. Gen. de Med.*, 1836, p. 339), Delasiauve (*Loco cit.*, p. 65) and Hasse (*Krankheiten des Nervenapparates*, 1855, p. 251), the epileptic cry, in the most complete cases of epilepsy, may not exist. I have witnessed two fits of epilepsy in which the most violent convulsions and a complete loss of consciousness, followed by coma, took place without cries. Is the loss of consciousness the first symptom? Most of the principal writers, who ignore the power of the reflex actions, consider the cry as a proof of feeling : surprise, according to Bean; surprise and pain, according to Herpin (*Loco cit.*, p. 477) ; surprise, convulsion and pain, according to Delasiauve

(*Loco cit.*, p. 77), and they admit, therefore, that the loss of consciousness is not the first symptom, at least in most cases. Billod attributes the cry to the convulsive spasm of the laryngeal muscles, and to a convulsive expiration (*Annales Méd. Psychol.*, Nov. 1843). According to him, the loss of consciousness preceees the cry, which is not a symptom of surprise or of pain. Hasse considers the cry as being probably the result of a reflex action (*Loco cit.*, p. 251–2). I have tried to show elsewhere (*Exper. Researches applied to Physiol. and Pathol.*, New York, 1853, p. 54–5) that cries in animals or in children deprived of their brain, may be due to a mere reflex action; the vocal cords being contracted, and the expiratory muscles expelling quickly the air contained in the chest, the sound which we call a cry is produced. In epilepsy, the loss of consciousness, which is equivalent to the loss of the brain, allows a cry to take place by reflex action. In the most complete and violent fits of epilepsy, we think that the first phenomena are almost always 1st, the contraction of the blood-vessels of the face, which causes the paleness, noted particularly by Prof. Trousseau, by Delasiauve and by Dr. Bland Radcliffe; 2d, the contraction of the bloodvessels of the brain proper, which causes the loss of consciousness. The cry, either at the same time, or immediately after, is produced by the spasmodic contraction of the expiratory muscles driving the air forcibly through a contracted glottis. At the same time, also, almost always some muscles of the face, of the eye, and of the neck contract. Sometimes, also, the spasm extends at once to the muscles of the upper limbs, and afterward to the whole body. All these phenomena are sometimes produced at oncc, and are all the results of an excitation springing from some part of the excito-motory side of the nervous system. In other cases there is an evident succession in these phenomena; the paleness of the face and the loss of consciousness (both resulting from contractions of the bloodvessels) take place at first, with some spasmodic actions of the muscles of the eye and face, and then come the cry and the tonic contraction of the muscles of the limbs and trunk.

The following table will show how the principal phenomena are generated, *one by the other*, in the most common form of the violent and complete epileptic seizures.

| CAUSES. | EFFECTS. |
|---|---|
| 1. Excitation of certain parts of the excito-motory side of the nervous system. | 1. Contraction of bloodvessels of the brain proper and of the face, and tonic spasm of some muscles of the eye and face. |
| 2. Contraction of the bloodvessels of the face. | 2. Paleness of the face. |

| | |
|---|---|
| 3. Contraction of the bloodvessels of the brain proper. | 3. Loss of consciousness, and accumulation of blood in the base of the encephalon and in the spinal cord. |
| 4. Extension of the excitation of the excito-motory side of the nervous system. | 4. Tonic contraction of the laryngeal, the crevical and the expiratory muscles (larynismus and trachelismus). |
| 5. Tonic contraction of the laryngeal and of the expiratory muscles. | 5. Cry. |
| 6. Farther extension of the excitation of the excito-motory side of the nervous system. | 6. Tonic contractions, extending to most of the muscles of the trunk and limbs. |
| 7. Loss of consciousness, and tonic contraction of the trunk and limbs. | 7. Fall. |
| 8. Laryngismus, trachelismus, and the fixed state of expiration of the chest. | 8. Insufficient oxygenation of the blood, and general obstacle to the entrance of venous blood in the chest, and special obstacle to its return from the head and spinal canal. |
| 9. Insufficient oxygenation of the blood, and many causes of rapid consumption of the little oxygen absorbed, and detention of venous blood in the nervous centres. | 9. Asphyxia. |
| 10. Asphyxia, and perhaps a mechanical excitation of the base of the encephalon. | 10. *Clonic convulsions everywhere*, contractions of the bowels; of the bladder; of the uterus; erection; ejaculation; increase of many secretions; efforts at inspiration. |
| 11. Exhaustion of nervous power generally, and of reflex excitability particularly, except for respiration. Return of regular inspirations and expirations. | 11. Cessation of the fit; coma or fatigue; headache; sleep. |

We have but little to say in explanation of the above table, which only gives, as we hardly need to remark, a type of a complete seizure.

Writers on epilepsy are unanimous in considering the *fall* as due only to convulsions, while it is certainly, in a measure, the consequence of the loss of consciousness, which alone causes it in some cases of epileptic vertigo without convulsions.

We do not think that laryngismus in epilepsy has the immense importance given to it by Dr. Marshall Hall. In the first place, in persons in whom the reflex excitability is not increased laryngismus exists frequently, in whooping cough, in asthma, &c., without producing epileptic convulsions. In the second place, epileptic convulsions may exist before laryngismus (Hasse, *loco cit.*, p. 252). If, instead of saying that laryngismus is the essential cause of convulsions in a fit of epilepsy, we say that asphyxia, whether produced by laryngismus or by other causes, is the source of a certain part of the convulsions in the violent and conplete fits of epilepsy, we shall be much nearer the truth. If we say also that laryngismus is nothing but a spasm of certain muscles—spasm produced by a reflex action at the same time that there are other spasms in the bloodvessels of the brain proper, of the face, and also sometimes of the whole surface of the body, and in the muscles of the head, of the trunk and limbs, and that all these spasms are reflex contractions, due to the same excitation, we shall be much nearer the truth than by admitting Dr. Hall's views.

